# How does the use of humour in the UK ambulance service affect a clinician’s well-being?

**DOI:** 10.29045/14784726.2021.9.6.2.26

**Published:** 2021-09-01

**Authors:** Connie Lancaster, Peter Phillips

**Affiliations:** South Western Ambulance Service; Bournemouth University

**Keywords:** coping, humour, paramedic, resilience, well-being

## Abstract

**Introduction:**

Paramedics and ambulance staff face many different stresses in today’s UK ambulance service, with many having reported an effect on their well-being and mental health. Humour is widely used by staff as a coping mechanism, but little is known about this topic. This literature review aims to find out how humour is used and how it can affect clinicians’ well-being.

**Methods:**

A total of nine medical databases were searched for relevant literature – Cochrane, Scopus, CINAHL Complete, Science Direct, Medline Complete, Complementary Index, Academic Search Complete, Emerald Insight and Supplemental Index. Articles were included if they were published in 2005 or after, if they were a UK-based original study and if they studied humour in relation to paramedics’ well-being.

**Results:**

After limiters were applied, a total of 26 articles were found. Only four articles met all the inclusion and exclusion criteria. Two of the articles studied humour directly, whereas two found humour as a result of studying the resilience and strategies used to cope with the stresses of ambulance work. Four key themes were identified: different types of humour, the source and boundaries of humour, offloading and camaraderie.

**Conclusion:**

This review of the literature found that many in the ambulance community view their use and expression of humour as a positive coping strategy that helps them to relieve the stresses of the job. Further research is needed to investigate any negative effects that humour has on a clinician’s well-being and larger studies are needed to give a better representation of the ambulance community.

## Introduction

Since the publication of the Bradley Report, there have been increased expectations for paramedics to have a wider skill set, and to see and treat more patients (Bradley, 2005). This presents new challenges to paramedics that may influence their well-being. It is widely acknowledged that working within the ambulance service takes a toll on the well-being of staff (Johnson et al., 2005; Shepherd & Wild, 2013). Well-being is about how comfortable, healthy, happy and satisfied people are within their lives (Appleby, 2016) and negative effects on well-being can range from stress at one end of the spectrum to anxiety, depression and post-traumatic stress disorder (PTSD) at the other. The NHS reported that staff sickness due to stress was highest among ambulance staff (Health and Social Care Information Centre, 2014), which may be due to the high levels of stress experienced by ambulance staff. Mind found that 91% of ambulance staff have experienced stress and poor mental health (Mind, 2019) and although this figure may be higher due to the study being self-reporting, it is still an important figure to consider. Additionally, 10% of ambulance workers showed signs of clinical depression and the prevalence of PTSD was 22% (Bennett et al., 2004). Drewitz-Chesney (2012) reports that paramedics have the highest prevalence of PTSD out of all the emergency services.

Clinicians may have developed coping strategies, such as humour, in response to negative effects on their well-being (Rowe & Regehr, 2010).

Humour within healthcare is a controversial topic, partly because it can be easily misinterpreted and needs to be used in the right circumstances (Buxman, 2000; Caudill & Woodzicka, 2017). However, humour within healthcare is not as inconsequential as some may suggest: it humanises peoples’ experiences within care, especially in stressful situations (Dean & Major, 2008; Sliter et al., 2013). Studies have shown that when humour is used among healthcare employees, there is a positive effect on well-being (Dean & Major, 2008; Rowe & Regehr, 2010). Through two different studies that both used clinical ethnography, with observational field work and semi-structured interviews, Dean and Major (2008) found commonalities to determine the value of humour when used in different healthcare settings. They found common themes including relieving tension and humanising experiences of healthcare workers, both of which have positive effects on well-being. Rowe and Regehr (2010) explored previous studies on humour and the emergency services, including paramedics. They found that when humour was used it was a way of expressing socially unacceptable impulses, which would cause a cathartic effect. These in turn were shown to have decreased negative feelings and relieved stress – again, positively effecting the well-being of the emergency service workers.

Factors that have contributed to this positive effect range from benefiting social support, easing tense situations and managing emotions to improving communication. A well-known pioneer in humour research, Paul McGhee, states that although there is no substantial research to correlate with the use of humour and physical health, there is much more research to substantiate claims that humour has a positive impact on well-being (Papousek, 2018).

Currently there are no literature reviews on how humour correlates to a paramedic’s well-being. It is important to further our understanding of how and why humour is used and how it influences well-being. This review aims to answer the question: how does the use of humour in the UK (United Kingdom) ambulance service affect ambulance clinicians’ well-being?

## Methods

The PEO model was used (Table 1) and search terms were created with synonyms (Table 2). These formed the search terms for all databases.

**Table 1.** PEO model.

	Term
**Population**	Paramedic
**Exposure**	Humour
**Outcome**	Well-being

**Table 2.** Search terms and synonyms.

Term	Synonyms
Paramedic	‘Paramedic*’ or ‘ems’ or ‘emergency medical service’ or ‘prehospital’ or ‘pre-hospital’ or ‘ambulance’ or ‘emergency medical technician’ or ‘emt’ or ‘frontline’
Humour	‘Humour’ or ‘humor’ or ‘laugh’ or ‘laughing’ or ‘laughter’ or ‘jokes’
Well-being	‘wellbeing’ or ‘well-being’ or ‘stress’ or ‘worry’ or ‘depression’ or ‘anxiety’ or ‘PTSD’ or ‘post traumatic stress disorder’ or ‘emotion*’

Limiters were applied to form the eligibility criteria, therefore making sure the literature included was up to date and applicable. The first limiter was to make sure the articles were peer reviewed. This was important because peer-reviewed articles should be of a higher quality (Kelly et al., 2014). The date range was from 2005 to January 2021 when the final search was conducted. The year 2005 was chosen as the cut-off date due to the Bradley Report being published that year. This report marked a new era for the ambulance service and helped form the ambulance service we know today (Bradley, 2005; Woollard, 2006). In order to understand the literature included, the articles also had to be in the English language; the third limiter.

Nine medical databases were searched for relevant literature. These were: CINAHL Complete, Science Direct, Medline Complete, Complementary Index, Academic Search Complete, Emerald Insight, Supplemental Index, Cochrane and Scopus. The last search was conducted on 14 January 2021.

After screening the literature by both title and abstract for relevance, screening of the full text took place. Inclusion and exclusion criteria were used to screen articles (Table 3). In addition, any article that was full-text screened also had its references reviewed in order that any additional suitable studies may be discovered. However, no further studies were incorporated this way.

**Table 3.** Inclusion and exclusion criteria.

Inclusion	Exclusion
Original studies with primary research.	Any studies that took place outside the UK.
Any studies that included paramedics or ambulance staff.	Any studies that did not specify the type of emergency worker or did not talk specifically about paramedics or ambulance staff.
UK-based studies.	Any studies that did not find humour as an affecting factor on well-being.

**Figure 1:**
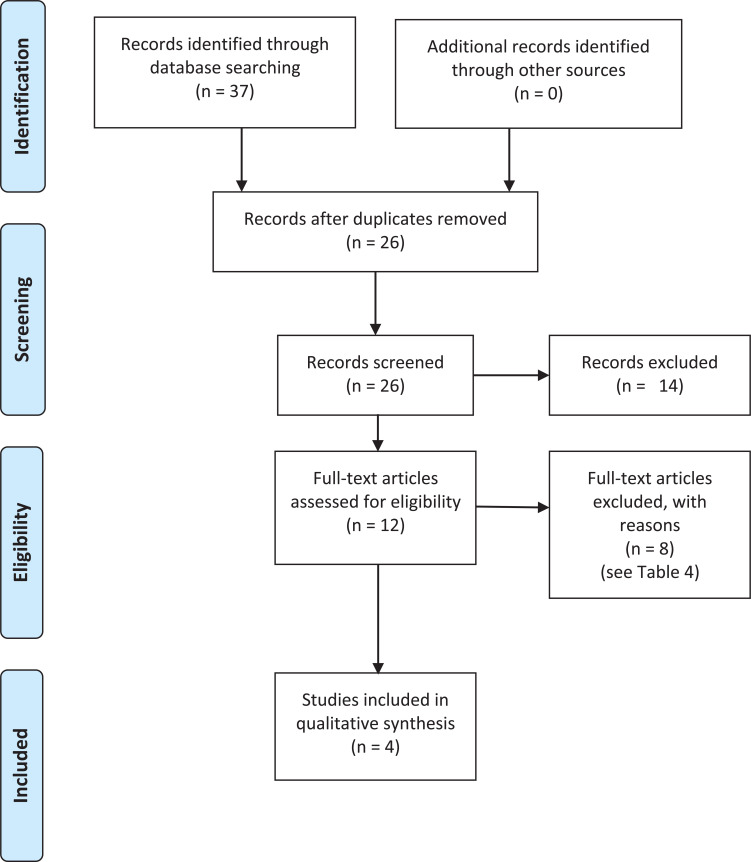
Figure 1. PRISMA flow diagram (Moher et al., 2009).

The first inclusion criteria set out that each article included had to be an original study, carrying out their own primary research. This was because articles that discussed previous research were at risk of discussing research that took place before 2005, the cut-off date. Also, reading and including others’ discussions and opinions may have introduced a risk of bias to this literature review. Therefore, to reduce bias, only original research was included. Any studies that took place outside of the UK were excluded so that the conclusion of this literature review could be better reflective of UK practice. Any articles that did not discuss humour and its effect on paramedics or ambulance staff were also excluded, as they were not relevant to the objective. To see the progression of the search strategy, refer to Figure 1. For the articles that were excluded after full-text screening and the reason why, refer to Table 4. To reduce bias further, the two authors conducted the search independently and then compared results to decide on which articles should be included. There were no disagreements over which articles should be included.

**Table 4.** Reasons for exclusions.

Article excluded	Reason for exclusion
Bochantin (2017)	This article did not specify that the public safety employees within the study were paramedics or ambulance staff.
Scott (2013)	This article did not mention humour as an effect on well-being.
Rowe & Regehr (2010)	This was not a study conducted in the UK and it discussed secondary research, not conducting any of their own primary research.
Williams (2013)	This article did not mention humour as an effect on well-being.
Mildenhall (2012)	This study was a literature review, therefore did not conduct their own primary research.
Roscoe (2017)	This was not a study that was conducted in the UK.
Kuiper (2012)	This was not a study that was conducted in the UK. It also did not specify that paramedics or ambulance staff were included in the study.
Christopher (2015)	This article discussed others’ secondary research and did not carry out any of their own primary research.

From these final articles, key information was extracted, as well as strengths and weaknesses (see data extraction table in Supplementary 1). By using the Critical Appraisal Skills Programme qualitative checklist (CASP, 2018), each article was appraised and given a rating of ‘high’, ‘medium’ or ‘low’, where a high rating has a low risk of bias and a low rating has a high risk of bias. All articles were of medium or high rating, therefore none were excluded based on risk of bias.

Thematic analysis was applied to the articles in a step-by-step approach (Nowell et al., 2017). After becoming familiar with the data, codes are raised into themes. These themes are then reviewed, and finally named, which then become the themes presented in the results and discussion (Nowell et al., 2017). These themes would be discussed by both authors to improve credibility (Nowell et al., 2017).

## Results

Four articles were included in the final review. Humour was discussed in all the final articles, but they all researched or discovered its use in different ways. Two of the articles studied humour directly, whereas the other two articles found humour as a result of their studies. Supplementary 1 shows the data extraction table with the key findings of each article. It also shows the CASP score for each article, which varied between 6 and 9, so all four articles were included in the final review.

The oldest of the four articles, conducted by Scott (2007), studied not only paramedics but also traffic officers and emergency department nurses. Through focus groups, Scott studied how each of these groups expressed humour within their roles involving sudden death work. For the purpose of this literature review, only the results highlighted as paramedics were included: only three paramedics took part. The study conducted by Charman (2013) took a similar approach to Scott and investigated directly how ambulance staff and police use humour. This study was conducted in an informal setting, consisting of 45 semi-structured interviews, 22 of which were by different grades of ambulance staff (paramedics, technicians and emergency care assistants). Again, like the study conducted by Scott, only the applicable responses identified have been included in this review: those highlighted as ambulance staff.

Humour was not always studied directly. Williams (2012) and Clompus and Albarran (2016) conducted studies on how ambulance staff deal with challenges at work. As a result, they found that humour was a type of coping mechanism. Williams (2012) studied, through semi-structured interviews, how eight undergraduate paramedic students dealt with the different emotions they may face while on placement. The final study carried out by Clompus and Albarran (2016) also had a small sample study of seven participants, five of whom were paramedics. They described a robust qualitative psycho-social methodology. There were two phases to their methodology: biographical narrative interviewing (phase 1) and semi-structured interviews (phase 2).

## Discussion

There were four different themes that became evident in these articles: the different types of humour expressed; how humour affects camaraderie; the source and boundaries of humour; and how humour may help with offloading.

### Different types of humour

It is widely acknowledged that humour is subjective, with each person finding different things humorous (Dunbar et al., 2016). There are many different types of humour and the study conducted by Scott (2007) identified seven categories. One category, ‘quick-witted quips’, was highlighted as the most commonly expressed by ambulance staff. Although ambulance staff also showed expressions of the other categories of humour, those categories were more commonly used by the nurses and traffic officers.

With such a small sample size of only three paramedics participating (Scott, 2007), it is unlikely to have given a true representation of the wider ambulance service and the type of humour they use. It was also a very localised study, meaning that it is important to take into consideration the local factors that might affect their type of humour. In one part of the UK, cultural cues and social boundaries can be very different from another part of the country, therefore potentially affecting the type of humour expressed (Wilkie, 2020). A study conducted in Glasgow looked at how where a person lives affects their mental well-being (Jones et al., 2014); similarly, it may influence how people perceive humour and how they express it. Scott (2007) discussed how tragic events that have occurred affect emergency personnel’s expression of humour and how it may be a darker humour expressed. With an increase in terror attacks and violent crime occurring, particularly in locations such as cities (Stripe, 2020), it is highly likely that the well-being of ambulance staff will be affected, especially those who have experienced these events first-hand and have been affected by them, and this could also influence how they perceive and express their humour. These are valid considerations when looking at the location of the participants. It would have been more beneficial for this study, as well as the others, to have taken place across different parts of the UK. This would have given a wider perspective of expression of humour among ambulance staff and minimised selection bias, a type of bias that can be introduced when selecting participants for a trial or study (Henderson & Page, 2007).

Scott (2007) suggests that the use of quick-witted quip humour may be the most commonly expressed type of humour because it becomes second nature to staff. This type of humour is quick and doesn’t take much thought, similar to subconscious thinking that happens automatically. It may evolve as staff want to see the lighter side of stressful situations, which results in quick-thinking jokes and foolish jesting with crewmates. This, in turn, can lead to camaraderie.

### Camaraderie

A strong theme that emerged from reviewing the final articles was camaraderie, with three of the four discussing it. Teamwork and good communication are recognised traits that make a good paramedic and help to improve patient care (Naya et al., 2014; Williams et al., 2013). With camaraderie, teamwork can improve as staff feel they can trust and rely on their colleagues, in turn improving patient care. This then has a positive impact on staff’s mental health and well-being, as well as being perceived as a type of social support (Rego et al., 2009).

Although Scott’s research found camaraderie to be a consequence of when staff made jokes and laughed together, Clompus and Albarran (2016) discovered camaraderie in other ways. They discovered that the participants felt that humour was a release and that releasing their emotions through humour allowed for camaraderie through the support they received socially from their crewmates. This study can be considered reliable due to its robust and descriptive methodology. It had a two-stage methodology. The first stage allowed for the participants to tell their experiences freely without prompting. This meant that the risk of interviewer bias was reduced. By not prompting the participants, they were not guided by what the interviewer may have wanted to hear. The second stage employed a semi-structured interview approach, which may have then introduced some bias from the interviewer. However, this aspect is also tackled as they recognised the relationship between the interviewer and the participant and the potential effects it may have. They have used the Free Association Narrative Interviewing procedures which allow for more interpretation of participant’s experiences, not just considering their words but also the tone of their voices and any feelings that may have arisen (Given, 2008). All the data were rigorously analysed, and discrepancies discussed. This type of methodology aims to overcome the challenges and biases of qualitative research, and for that reason this study’s results are reliable.

Another study suggested that humour strengthens employee relationships, which then leads to improved customer service and satisfaction and staff well-being (Mathies et al., 2016). Although not a healthcare-focused study, it still shows how humour is important within staff relationships. Charman (2013) agreed and additionally suggested not only that humour brings a sense of camaraderie, but also that the exposure the staff have is often the root of the humour. It is important to consider this aspect, as this exposure has been suggested in some of the other literature to be the source of humour in the ambulance service (Scott, 2007).

### Source and boundaries of humour

Charman (2013) explored the sources of humour, and although this study investigated police officers as well as paramedics, there were clear distinctions as to which experiences were from paramedics. This study had the largest sample group, consisting of 22 paramedics. This is a much better sample size as it gives a broader spectrum to the results and provides a better representation of the paramedic community. However, when looking at this study, it is important to consider the setting in which it took place. It carried out its semi-structured interviews in the crew rooms of participants, including some elements of ethnography, although a limitation of the article is that the methods are not as clearly described as they could be; the confidentiality of the participants must then be considered as it may have affected their answers. For example, participants may not have given honest answers due to fear of being overheard by those not involved in the study. Also, the article never states if it gained any ethical approval, which may make the results of this study not ethically reliable.

Nonetheless, Charman (2013) does raise some important aspects which some of the other literature agrees with. The most common finding was that the source of most jokes and expressions of humour is other members within the ambulance staff. This was noted to be most prevalent when the participants were on their breaks, to help them relax. The second most common source of the humour was the public. The patients that the ambulance staff attended to were often the source of jokes and humour.

However, when it came to this source of humour it was clear that there were clear unwritten boundaries set by the staff. These included not making jokes about children or terminally ill patients, as well as keeping jokes between colleagues they know well and not retelling jokes to friends and family outside of the ambulance service. These boundaries appeared to be of importance to all the participants, with boundaries being discussed in all the studies (Charman, 2013; Clompus & Albarran, 2016; Scott, 2007; Williams, 2012). They are seemingly set to keep respect for the patients and their families. This respect is important as it is one of the core values set out by the National Health Service (NHS), and therefore needs to be adhered to (Department of Health and Social Care, 2019). Humour at the expense of the public may cross the line regarding respect and so any boundaries set out to try and keep this respect are needed. Whether or not these boundaries do enough to upkeep this respect is subjective to each person.

The final source of humour identified by Charman (2013) was other emergency services, most commonly the fire service. It was highlighted that any humour directed at the fire service was always friendly. Professional jealousy may be a reason behind this source of humour. Many of the participants interviewed stated that the ‘banter’ around the fire service was mainly due to the fire service sleeping on night shifts. This may cause some jealousy as it is not often that ambulance staff manage to sleep on a night shift, therefore generating some jokes around the subject.

### Offloading

Of all the themes that were drawn from the final articles, offloading was mentioned throughout all the papers. In all the articles, participants saw humour as a way to ‘offload’ their worries and stresses as well as a way to see the lighter side of a situation.

The study carried out by Williams (2012) on the resilience strategies of student paramedics found that humour was unanimously recognised as a coping strategy. There is a strong link shown in other studies between humour and an increase in resilience (Kuiper, 2012). Williams found that by using humour, participants were able to express their emotions, especially in emotionally challenging situations, and to help see a less emotionally draining side of a situation. This allowed them to release any worries and stresses they had. Although this study included student paramedics, it is important to consider the effects that humour has on those who are essentially the future of the ambulance service. Williams also had a small sample study of only eight participants and the lack of diversity of the participants was never recognised. In order to obtain a good representation of the paramedic community’s relationship with humour, it is important to have diversity throughout the chosen sample. Ideally, an equal number of male and female participants, with a variety of ethnicities and cultures, would be included in the sample, as all may perceive humour in different ways according to their sex, ethnicity and culture.

Although these factors were not taken into consideration when this study took place, the other final articles agree that all their participants felt that humour was a good way to release their emotions. They also perceived it as a form of informal peer support and that it acted as an informal debrief after jobs (Charman, 2013; Clompus & Albarran, 2016; Scott, 2007).

Another study that was excluded from this review of the literature, due to it studying solely firefighters, also found that humour is a positive coping mechanism to offload stresses and to help staff move on since, like paramedics, firefighters come across many stressful situations. The study even suggested that future training on how to cope with the stresses of the job should focus on using humour (Sliter et al., 2013).

## Limitations

One limitation of this review is the number of databases that were able to be accessed. If more databases had been available, there is a possibility that more eligible articles would have been included and the findings could have altered. Also, it did not consider grey literature as this was not feasible within the scope of the review.

## Conclusion

While completing this review, some gaps in research have been identified. There is very little UK-based research completed on the direct effect that humour has on the well-being of ambulance staff and even less on solely paramedics’ well-being. Future research with a larger and more diverse sample would be beneficial in representing the UK paramedic population. There is also a complete lack of research regarding any negative effects that the use of humour may have on a UK paramedic’s well-being. These are both areas that need to be researched further, to come to a more accurate and representative conclusion.

It could be suggested that the use of appropriate humour should be encouraged between staff to allow for the relief of stresses relating to front-line ambulance work, and to strengthen the camaraderie between staff. However, the classification of appropriate humour is very subjective, which may pose challenges. A literature review completed regarding nurses and the use of humour suggested that they should use an evidence-based style when using humour in the workplace (McCreaddie & Wiggins, 2008). This approach to using humour within the workplace could improve the well-being of staff and therefore also the care that patients receive. However, if this were to be encouraged, it should be clear that in doing so, the unwritten boundaries that staff expressed and that were discussed in this review as well as other evidence should be used to underpin the use of humour.

The four themes identified all link together, with the source and boundaries of the humour expressed creating the ‘quick-witted quips’ type of humour which brings staff together in a sense of camaraderie. This then helps staff to offload their worries and stresses, as they feel they can trust their crewmates with any worries they have. Overall, the current literature suggests that many ambulance staff feel that the use and expression of humour helps them to cope with the different stresses and situations that front-line ambulance work poses. Therefore, humour has a positive impact on well-being and a cathartic effect on staff working within the UK ambulance service.

## Author contributions

CL devised the review topic and question. CL and PP both developed the search criteria and independently ran database searches. CL and PP both decided on final articles. CL wrote up the results and discussion. CL penned the majority of the article, with some input from PP. PP acts as the guarantor for this article.

## Conflict of interest

None declared.

## Ethics

Not required.

## Funding

None.
